# Exploring the Interactions between Housing and Neighbourhood Environments for Enhanced Child Wellbeing: The Lived Experience of Parents Living in Areas of High Child Poverty in England, UK

**DOI:** 10.3390/ijerph191912563

**Published:** 2022-10-01

**Authors:** Marcella Ucci, Adriana Ortegon-Sanchez, Naomi E. Mead, Catherine Godward, Aamnah Rahman, Shahid Islam, Nicholas Pleace, Alexandra Albert, Nicola Christie

**Affiliations:** 1 UCL Institute for Environmental Design and Engineering, The Bartlett Faculty of the Built Environment, University College London (UCL), London WC1H 0NN, UK; 2 Centre for Transport Studies, Department of Civil, Environmental and Geomatic Engineering, University College London (UCL), London WC1E 6BT, UK; 3 Bromley by Bow Centre, St. Leonard’s Street, London E3 3BT, UK; 4 Department for Transport, Great Minster House, 33 Horseferry Road, London SW1P 4DR, UK; 5 Bradford Institute for Health Research, Bradford Teaching Hospitals NHS Foundation Trust, Bradford BD9 6RJ, UK; 6 Centre for Housing Policy, University of York, Heslington, York YO10 5DD, UK; 7 Thomas Coram Research Unit, University College London (UCL), 27-28 Woburn Square, London WC1H 0AA, UK

**Keywords:** housing, children, health, inequalities, neighbourhood, lived experience

## Abstract

Children’s health can be affected by the interrelated characteristics of the physical and social environment where they live, including housing quality, neighbourhood characteristics and the local community. Following a systems-based approach, this exploratory project sought to understand how the needs and aspirations associated with the home environment can work in synergy with, or be exacerbated by, other aspects of the local area. The study recruited parents of children aged 2–12 years old from two local authorities in England with high levels of child poverty: Tower Hamlets in East London, and Bradford District in West Yorkshire. Thematic analysis of participant interviews highlighted ten themes and opportunities for improvements. The evidence presented in this research emphasises how environmental quality issues within and outside the home, compounded further by delays in repairs and reduction in service standards, as well as affordability issues, are likely to deeply affect the wellbeing of an entire generation of disadvantaged children whose parents can feel disempowered, neglected and often isolated when attempting to tackle various dimensions of inequalities. Interventions which can improve the quality of housing, and access to space and services, are urgently needed, including initiatives to support and empower families and local communities, especially those prioritising opportunities for action.

## 1. Introduction

The significance of housing for health and wellbeing is well established—yet its impact on children specifically is relatively less understood [1]. Whilst housing is considered an important social determinant of health, guidelines and recommendations for ‘healthy homes’ often do not consider children specifically and focus especially on building-level hazards such as poor indoor air quality or risk of falls and accidents [2], with limited focus on aspects such as access to play/green areas [3]. Although much evidence on housing and health covers environmental hazards caused by disrepairs, poor quality design/construction, and outdoor hazards (e.g., ambient pollution), other factors are important for children’s wellbeing, namely security and stability of tenure, as well as the extent to which the ‘home’ meets the needs of children and their families (e.g., lack of space, children with disabilities) [4]. Whilst some aspects of housing such as tenure and/or dwelling type [5] could be considered a proxy for other social determinants of health, evidence shows that poor housing quality is associated with poorer child health, after controlling for factors such as affordability and neighbourhood safety [6]. Furthermore, whilst some research exists on neighbourhood quality and on the role of social capital/networks on health and on inequalities [7,8], fewer studies seek to understand the potential for interaction—whether negative or positive—between the home environment and the local neighbourhood. 

The neighbourhood’s physical environment, in combination with perceived safety of the local area, are acknowledged as important social determinants of health in early childhood [9]. For example, access to green space can positively impact children’s mental wellbeing [10] and youth development [11]. Some studies have also explored whether the combination of high-deprivation home and neighbourhood environments may exacerbate negative impacts upon children’s health, development and physical activity or sedentary behaviour [12]. Research also shows that in deprived urban areas, adult mental wellbeing is associated with the quality and aesthetics of housing and neighbourhoods, as well as with feelings of respect, status and progress related to how places are created, maintained and talked about [13]. In cities, family outings, which are important for family ties and growing up, have been found to differ within the same neighbourhoods depending on socio-economic status [14]. Data from the UK Household Longitudinal Study found that housing type, tenure, cost burden and ‘desire to stay in own home’ were associated with biomarkers of infection and stress in adults [15]. This evidence re-enforces the need to consider housing, and neighbourhoods, as a psychosocial as well as a physical environment that can affect health and wellbeing. It should be noted that there is potential for feedback loop mechanisms between psychosocial and physical stressors, with some authors arguing that individual-level and placed-based psychosocial stressors (such as limited access to services) may increase susceptibility to environmental hazards at relatively lower doses, partially explaining some disparities in maternal and child health [16]. 

The local context has been identified as one of four ‘pillars’ for conceptualising housing and health disparities, alongside housing conditions, housing costs, and residential stability [17]. Therefore, holistic and systems-based approaches [18] are needed to explore the links between psychosocial and environmental stressors and, more broadly, to further understand the complex and dynamic ways through which ‘housing’ and ‘neighbourhoods’ individually and together interact to affect children’s health. On the other hand, there is scarcity of research on whether the lived experience of the home environment is affected by the local area (and vice versa) and thus in turn how these relationships may affect health behaviours and outcomes in children. Such relationships are likely to be shaped by the unique combination of the local neighbourhood and housing physical environments, the local community, and the availability of services and facilities in the area. It is thus important to consider the question of possible synergies between housing and the local area from a place-based perspective—i.e., to what extent ‘good housing’ may be offset by deficiencies in the local area related to environmental characteristics, community cohesion and partnerships between individuals, communities and stakeholders. Furthermore, previous studies have argued that whilst much research on health-supportive built environments is centred around the quantitative paradigm of epidemiological studies, qualitative research drawing on the ‘lived experience’ of the local community can be fundamental in determining suitable interventions that match levels of community readiness before implementing any changes [19,20]. 

Taking the UK as a case study, significant inequalities exist in housing quality, affordability, and security of tenure [21]. Whilst overcrowded dwellings in England are more prevalent in ethnic minority households and amongst renters [22], there is limited research on whether access to green space and/or play facilities may, at least in part, alleviate the negative impacts of overcrowded housing on children and their families. The issue of housing quality is especially important when considering the impacts of the COVID-19 pandemic. In particular, how children may have been impacted by lockdown policies which severely restricted access to facilities beyond the immediate local areas and which imposed longer time spent in the home environment, which in turn increases exposure to housing-related hazards. Indeed, some authors have argued that the pandemic has put into sharp focus poor quality housing in England [23]: for example, the statutory definition of overcrowding means that a single parent with two children under the age of ten could live in a studio flat of just 20 square meters without being considered as living in overcrowded conditions [24]. Surveys carried out during the onset of the COVID-19 pandemic in England found high prevalence of poor quality and/or overcrowded housing in families living in a multi-ethnic and deprived city (Bradford), with housing insecurities especially common for those who were self-employed, not working or unemployed [25].

Following a systems-based approach and focusing on children’s health and wellbeing, this exploratory study seeks to understand how, within the context of deprived areas, the needs and aspirations associated with the home environment can work in synergy with other aspects of the neighbourhood or can in fact be exacerbated by the lack of local infrastructure, assets or services. The research explores the lived experience of parents living in two areas with high levels of child poverty in England (UK): Bradford Metropolitan District in West Yorkshire and Tower Hamlets in London. The study is part of ‘ActEarly’, a wider five-year research programme that focuses on upstream early life interventions to improve the health and opportunities of children living in deprived areas, whereby Bradford and Tower Hamlets are City Collaboratory testbeds [26]. Overall, this mixed-methods research aims to explore parents’ perceptions about the quality of their home and local outdoor environments as well as community assets, focusing on the potential for complementarity between housing and neighbourhood-level aspects, which could support (or hinder) their children’s health and wellbeing. Ultimately, the research seeks to identify potential opportunities for interventions to enhance children’s wellbeing in deprived areas, specifically via leveraging positive synergies between ‘inside’ and ‘outside’ the home environment and targeting aspects that prevent such synergies from being realised.

## 2. Methods

### 2.1. Settings and Participants

The study is set in Tower Hamlets (London) and Bradford Metropolitan District in West Yorkshire, two local authorities in England (UK) which are both characterised by high levels of child poverty and an ethnically diverse population, but differ in aspects such as population density and built form, with Tower Hamlets being more densely built and experiencing gentrification in some areas, resulting in higher housing prices and costs. 

Specifically, according to the Census 2011, in Tower Hamlets, the two largest ethnic groups in the borough are White British (31%) and Bangladeshi (32%) [27], whereas in Bradford, 66% of the population self-identified as White British and 20.4% self-identified as Pakistani [28]. In 2019, the population density (people by square km) in Tower Hamlets and Bradford was dramatically different, respectively, 16,237 and 1475 [29]. On the other hand, in the financial year ending 2020, Bradford was amongst the 20 local authorities in the United Kingdom with the highest proportion of children aged under 16 in ‘Relative low-income families’ (37.6%) and in ‘Absolute low income families’ (32.3%) [Definition of *Relative Low Income*: A family in low income Before Housing Costs (BHC) in the reference year. A family must have claimed Child Benefit and at least one other household benefit (Universal Credit, tax credits or Housing Benefit) at any point in the year to be classed as low income in these statistics. Definition of *Absolute Low income*: A family in low income Before Housing Costs (BHC) in the reference year in comparison with incomes in financial year ending (FYE) 2011. A family must have claimed Child Benefit and at least one other household benefit (Universal Credit, tax credits or Housing Benefit) at any point in the year to be classed as low income in these statistics]. The corresponding proportions in Tower Hamlets of children aged under 16 living in low-income families were, respectively, 28% (Relative) and 22.1% (Absolute), which was still higher than the England mean (19.1% Relative and 15.6% Absolute) [30]. Conversely, when considering child poverty after housing costs, in the fiscal year ending in 2020, 56% of children younger than 16 years old in Tower Hamlets lived in households with incomes below 60% median income, whereas in Bradford the percentage of children living in poverty did not change substantially (38%) compared to that before housing costs, suggesting that high housing costs in Tower Hamlets may be a contributing factor to child poverty [31]. 

In partnership with community-embedded organisations in Tower Hamlets and Bradford, the study aimed to recruit approximately 12 households (with a child aged 2–12) across two deprived neighbourhoods with different provision of local amenities (e.g., green spaces/playground, community centre, etc.) in each site. Neighbourhood selection was initially carried out based on the community researchers’ knowledge of the area and connections within the local community, considering also aspects related to housing quality and facilities in the local areas. Specifically, a purposive sample approach was utilised [32], designed to explore the lived experiences of children and families potentially facing housing-related issues, built co-productively with local community organisations who also played a key role in the participant recruitment and subsequent interviews, through a community research model [33]. The research had an explicit focus to include participants who have had minimal engagement in research studies and therefore can be described as seldom heard. Through the application of co-productive research principles where members of the target community became partners in research [34], we were able to leverage the experiential knowledge and pre-existing social networks of established community gatekeepers for inclusive recruitment. 

Participants were recruited amongst those known to the community researchers as living in the target sites. Although home ownership was not an exclusion criterion, most participants were social or private renters. The areas which were targeted in Bradford (Manningham/Girlington and in Keighley) because they were known to the researchers as having poor housing quality and high levels of deprivation also had a higher prevalence of private rented properties than the areas targeted in Tower Hamlets (Watts Grove and Bromley by Bow). In the latter, there was a greater prevalence of social housing (mainly purpose-built flats, mid to high-rise blocks) compared to the Bradford sites, where the prevalent housing type was terraced housing or low-rise, purpose-built flats. In Tower Hamlets, the Watts Grove area included a substantial new development, in contrast with the Bromley by Bow area, which comprises a more established community and variety in property ages. The sample in Bradford was more ethnically diverse than in Tower Hamlets, which may be due to a variety of reasons (e.g., differences in the local areas) but also the research team in Bradford was able to carry out interviews in the participants’ first language if preferred: Urdu, Punjabi and Roma (Slovakian).

**Figure 1:**
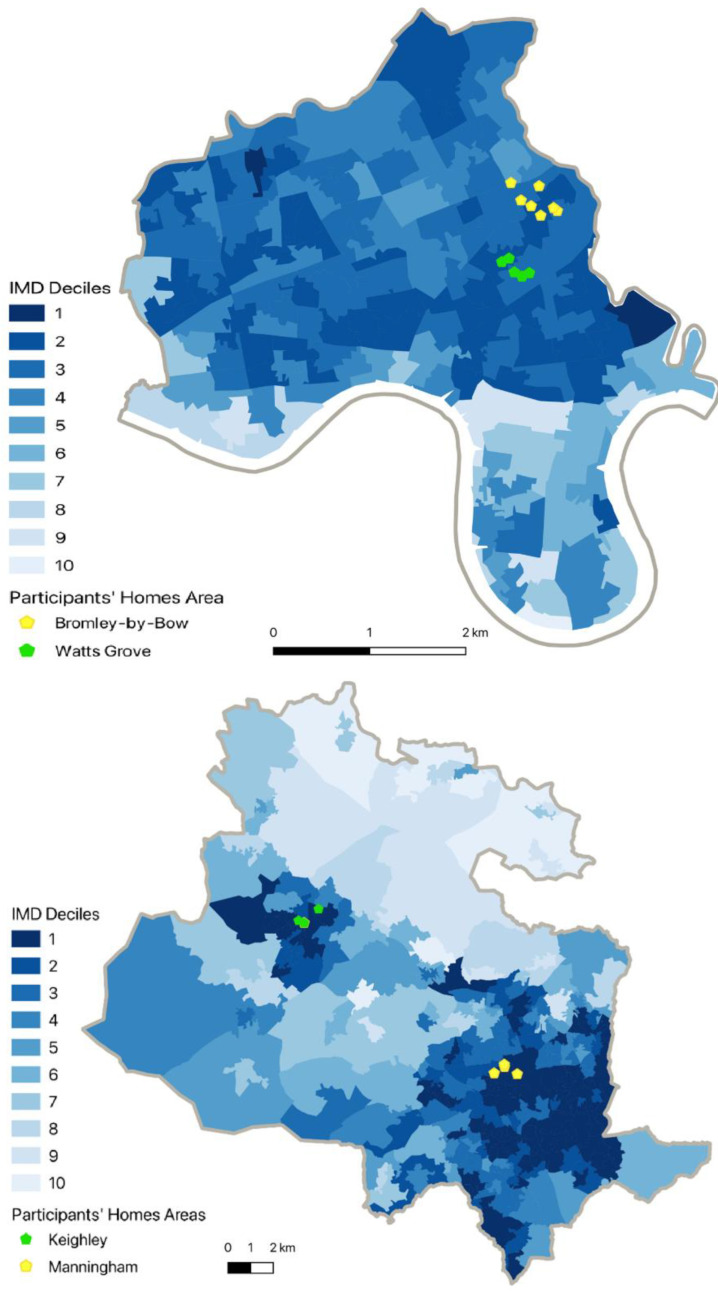
Location of interviewees’ homes within each local authority and IMD deciles (Tower Hamlets, (**top**); Bradford, (**bottom**)).

The maps in Figure 1 present the location of the interviewees’ homes (when available), the study areas and the 2019 IMD (Index of Multiple Deprivation) decile for the Lower Layer Super Output Areas (LSOA) where the homes are located [35].

### 2.2. Data Collection and Analysis 

Data collection took place over the summer 2021. Each participant was invited to join a 1 h interview with a community researcher, regarding their lived experience and perceptions of indoor and outdoor environment aspects. Most interviews were conducted online or on the phone, depending on participants’ needs and IT proficiency, as well as to account for any relevant COVID-19 infection risks or restrictions. At the beginning of the interviews, participants had an opportunity to ask any questions on the information sheet and consent form previously received, and verbal consent was recorded accordingly. As part of the description of their lived experience and to enrich the discussion during the interview, participants were also invited to collect and share, prior to the interview, photos of their home and local environments (things they liked or disliked) with no identifiable elements. However, this aspect was optional, to avoid excluding participants based on their ability or willingness to take or share photos. A semi-structured interview topic guide included questions about:

1. *General information* (e.g., how many children in the household, if any children with pre-existing health/wellbeing problems).
2. *Aspects of the local area and/or community* which support, or hinder, child’s health and wellbeing.
3. How the *home environment* supports, or hinders, child’s health and wellbeing. Within this context, related aspects such as the relationship with the landlord (if applicable) and cost of living were covered.
4. *Synergies or conflicts* between home and local environments
5. Reflections on *changes/improvements*: what they would ideally change in their home and/or local area and community to improve child’s health and wellbeing.

Prompts were used, when needed, for aspects within points 2 and 3 above, to ensure similar aspects would be considered across interviews. These included the key factors which previous research indicated as relevant, such as quality and provision of parks, play areas or child-friendly activities, or housing quality/repairs issues (e.g., dampness, heating). Since the interviews were carried out in summer 2021, although the research was not directly related to the impacts of the COVID-19 pandemic and associated lockdowns, interviewees were also asked if any aspects they had discussed in the interview had changed throughout the COVID-19 pandemic, and if so how. The questions did not refer to specific definitions of health and wellbeing; however, interviewees were instructed to prompt, when required, about any ‘*aspects that help your child to develop, to play, to learn, to thrive, to have good health*’. Participants with more than one child aged 2–12 were instructed to reply to questions in reference to one child only, or all/some of the children as they preferred, depending on the nature of the question(s). Most participants discussed all of their children at different stages of their interview. 

In collaboration with the Bromley by Bow community research team, interviews from the Tower Hamlets sites—where data collection was first completed—were analysed and thematically coded to identify emerging topics [36]. The interviews from the Bradford sites were then considered against the initially derived codes and themes, with an aim to understand how these related to the Bradford participants, and if further/different aspects emerged. 

After the interview, participants were also asked to complete a questionnaire online. In order to avoid overly long interview time which may have affected participant availability, the online questionnaire could be completed in their own time but with an option to request support from the community researchers for completing it if needed. The HAPIE (Health and Place Interventions Evaluation) tool was developed in the context of the ActEarly project [26] to evaluate people’s perceptions of their streets, neighbourhoods and home environments in relation to their wellbeing and self-reported health. The tool is structured in six parts:

- Part 1 Your experience of the street, drawing from the Healthy Streets framework, considers the assessment of street conditions, including experiences while on the street
- Part 2 Your activities and wellbeing: measures of mental wellbeing (WEMWEBS 7-item [37]) and reported physical activity
- Part 3 Your area: perceptions of the area
- Part 4 Your household: housing environment questions (residents, type of property, tenure, etc.) and access to car/bicycle
- Part 5 About you: demographics, post-code and reported health
- Part 6 Your views: open text questions

## 3. Results

### 3.1. Participant Details and Questionnaire Results

Overall, thirteen participants were interviewed in Tower Hamlets and nine in Bradford, some also providing photos, which were mostly of the outdoor environment. Most interviewees fully completed the online questionnaire (twelve in Tower Hamlets, and seven in Bradford). Table 1 shows participant characteristics including demographics and some other relevant aspects covered in the HAPIE tool. It shows that the majority of our participants were females self-identifying as ‘Asian or Asian British’, self-reporting opposing levels of their (adult) wellbeing on the WEMWEBS scale. Specifically, seven out of thirteen participants in Tower Hamlets scored ‘Low’, with the remaining five scoring ‘High’, whereas four out of seven participants in Bradford scored ‘Low’, with the remaining three scoring ‘High’. The vast majority of participants felt they did not have enough living space, and many reported difficulties with finances although some preferred not to disclose this aspect. 

### 3.2. Thematic Analysis 

**Figure 2:**
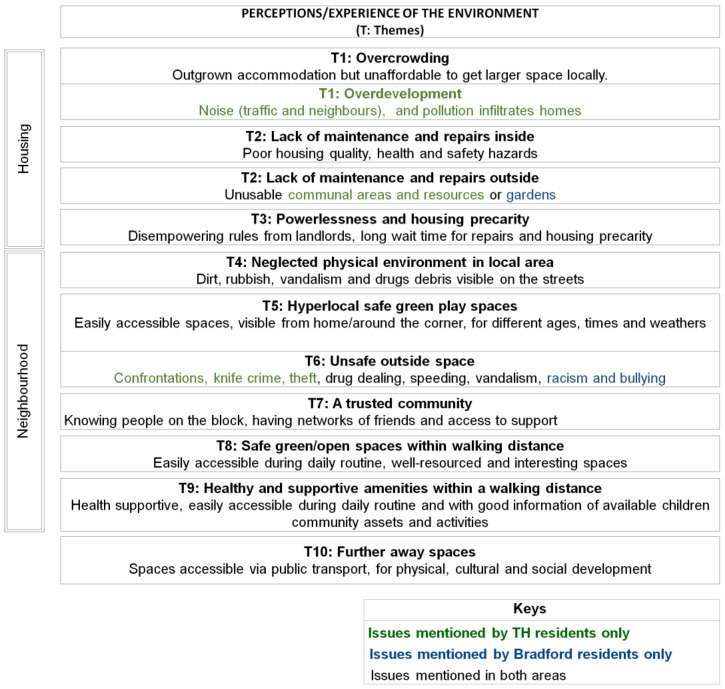
Themes—Perception and experiences of the environment.

Overall, ten emerging themes were identified from the thematic coding analysis across both sites. This section presents a narrative synthesis of the themes as well as key quotations from the interviews. Two researchers (AO) and (NC) carried out the initial analysis. The semi-structured topic guide questions acted as a priori headings and were refined and developed based on the participant narratives. Codes were developed independently by the two researchers using Nvivo 11 (QSR International) from the first few transcripts, and then coding books were shared; coding was fine-tuned after discussion though there was a high level of agreement between coders. The themes describe participants’ perceptions and experiences of their environment starting from inside their home environments, advancing to their immediate outside space (communal areas or gardens) and finally reaching other outside spaces, such as the spaces and amenities in the neighbourhood and in the wider local area. Figure 2 presents a summary of the ten emerging themes. It should be emphasised that the mechanisms through which the themes impact children’s wellbeing are heavily interconnected, as explored in the discussion section. 

#### 3.2.1. Theme One: Overcrowding and Overdevelopment in the Area

For study participants in Tower Hamlets (TH), the greatest concern inside the home was overcrowding; however, this concern was mentioned less by residents in Bradford who mostly lived in houses as opposed to flats. In TH, ten interviewees reported that they did not have enough living space, and some mentioned they had outgrown their accommodation some time ago but could not afford to achieve more space locally. Left with the decision to relocate for more space or maintain their children’ routine and networks, they prioritised the latter, considering it on balance as the better option for their children’s wellbeing.

*‘* *One of the biggest concerns in our area is overcrowding. […] children just not having enough space for study, for play within their homes.’*

(TH11)

Overcrowding forced participants to try to use space flexibly for multiple needs, but not always successfully. This particularly impacted on children’s quiet or private space and time with families, a major concern for interviewees.

*‘I’m sharing a bedroom with my daughter, that’s one thing I would change.’*

(TH2)

*‘We basically live, eat, and entertain in the living room.’*

(TH9)

In some cases, parents felt that having enough space within the dwelling was prioritised above gaining outside private space:

*‘I would give them space. Not for the garden, I don’t mind. However, for her to go into her own room and play. Even space to have a dining table and chairs to eat together’.*

(TH8)

In Bradford, a single mother with three children (4.5, 10 and 14 years old) mentioned they had two bedrooms, but they all slept in the same room: ‘[…] *because my eldest son has issues. I’m a single parent with three kids*’ (BD7). On the other hand, access to balconies (especially large ones) was considered very useful for those living in flats, and families with secure communal landings said their children were able to play there, using the space as an easy to access extension to their home. In TH, some participants also discussed how overdevelopment of the local area created air and noise pollution which impacted their home environment. 

#### 3.2.2. Theme Two: Lack of Maintenance and Repairs 

Study participants said that properties and the surrounding area were not maintained sufficiently well. Poor housing quality was described by one participant as limiting the children’s activities and not giving them opportunities for development. 

Inside the home, a slow or complete lack of repairs and upkeep created a reduction in quality of life and health risks such as increased risk of allergies due to dust, breathing problems because of damp and mould and, for some Bradford participants, safety hazards and exposure to vermin (e.g., rats, insects):

*‘Usually they’re* [the housing association are] *good but certain stuff they leave it too late before they can come because they don’t count it as an emergency. For instance, I’ve got a bathroom and toilet which is combined together, and I’ve got five people in one property so you can imagine how often the bathroom is being used. I was left without any bathroom lights for a good three weeks.’*

(TH2)

*‘In kitchen ceiling felt down, I already asked landlord to fix that and he said no, that not his responsibility … I am worried about kids to go to kitchen and I do not feel safe there myself … I do not have anywhere to cook and my cooker is not working.’*

(BD4)

*‘Within our block itself we’ve actually set up a residents’ action group because of the poor conditions that everyone is living in. […] there are many residents who are suffering with damp, with constant leaks. Very old building, the repair work is shoddy … it just doesn’t get seen to properly.’*

(TH11)

*‘They* [children] *are poorly all the time, the doctor gave me a letter because he (the child) got a chest infection, over and over again. Because of that the doctor gave us a letter to send to the council, they came to my house and took pictures and then sent them to the landlord and said to them they need to do the work in two weeks. They (the landlord) said to me that I need to leave in two weeks and get another house.’*

(BD7)

The lack of maintenance or upkeep of communal areas in TH was an ongoing concern for participants and was considered hard to solve; these issues included excrement in stairwells, lifts not working, and rubbish not being collected regularly enough (the most common concern participants raised)—with unpleasant smells and vermin (e.g., flies) reaching the home environment. Similarly, communal resources, important for physical and social development, were described as unusable due to a lack of repair or maintenance (e.g., the football pitch in need of repair and polluted canal). 

All participants in Bradford lived in terraced or detached houses and thus had no communal areas such as entrance lobby to staircase. However, rubbish and litter in their own gardens and the street outside their home was a common concern. Most participants considered that the amount of rubbish and the associated foul smell and vermin made their gardens unsafe for children to play in and unsuitable for anyone to spend time in. The issues with rubbish were thought to be caused by the local council not emptying the outside bins regularly enough and cleaning up the streets and by neighbours’ neglect, lack of respect for the environment and misbehaviour (sometimes people threw rubbish into others’ bins or even put old furniture out on the street). Some participants mentioned a need for better information regarding how to recycle and dispose large household items:

*‘I’d say my garden is not that friendly to be honest, because of all the smell, rubbish, all those things, I don’t think I’d feel happy for her to play out, I don’t even let her out into the garden most of the time because of all the smell and the rubbish and everything.’*

(BD1)

#### 3.2.3. Theme Three: Powerlessness and Housing Precarity 

In TH, parents felt unable to help fix problems in the physical environment due to landlords’ inflexible rules and a very cautious approach to health and safety (e.g., no washing to be hung out to dry on balconies, and no items in communal hallways). This was described by one participant as:

*‘… a daily battle with them actually. Because where do you keep wet mops if there’s no airing cupboard, if there’s no storage, and it’s winter?’*

(TH9)

Study participants felt that unnecessarily limiting rules (e.g., from the social landlord) needed to be relaxed; residents to be empowered and resourced to make some repairs themselves, as individuals and groups; and for the time to wait for landlord repairs to be reduced for all. 

In Bradford, housing precarity was raised as an issue; one participant described how the uncertainty about the chances of staying in the same house created anxiety, lack of trust and fear both for parents and children:

*‘I don’t trust nowhere where I live because of other places that I’ve lived. So I won’t, I won’t ever feel safe in my home, do you know what I mean?’*

(BD9)

The same participant also described how financial struggles associated with housing costs resulted in losing the home and the children:

*‘because of housing situation and we had no financial support, all our source of income as benefits had stopped and we had no money for rent, so the landlord threw us out of the property, basically we had nowhere to live and that was the main reason for them to remove children from our care.’*

(BD9)

In TH, a mother of four children living with her partner in a three-bedroom house explained that although they qualified for a four-bedroom dwelling, there were none available. Furthermore, they were offered and had to accept a higher rent than what they could truly afford, because they had limited housing options after becoming homeless previously:

*‘* *When we knew, we said to the housing officer that we are not taking this property. And he said ‘You don’t have any option because you are from homeless’. So we don’t have any options to get anymore property because we are from homeless. This is another thing. There’s so many confusing systems, confusion everywhere.’*

(TH12)

The quote above also emphasises how lack of information and understanding of services (e.g., housing/welfare), combined with being in a disadvantaged situation (e.g., homelessness) can result in powerlessness and a perception that services are not user-centred. 

#### 3.2.4. Theme Four: Neglected Physical Environment in the Local Area 

Study participants in TH talked about evidence of neglect of housing and the local area, including dirt, vandalism, and drug debris. Parents equated this neglect with a lack of care and felt that increased security and penalties to prevent anti-social behaviour were needed:

*‘One person puts out one piece of rubbish and then within hours it piles up and no one seems to care to think that that is not the right way to do it. […] It just seems like a neglected part of Tower Hamlets.’*

(TH11)

In TH, the neglect was said to come from multiple places—individuals, housing association waste collection schedules. There were also multiple people and organisations who could have an impact on the spaces, including the landlord, council, community, residents, and housing association. Therefore, exactly where responsibility lay was seen as complex and unclear. In addition, participants said that where there was clarity, those responsible do not always take action successfully (e.g., landlords).

*‘First thing the CCTV, they’re not doing anything about it. Secondly the dustbin, the smell, it’s not good for your health and they’re not doing anything about it.’*

(TH2)

Parents in both sites described how hazards above heavily affected their families, adding to the burden of daily living and setting an unwelcome standard of behaviour for their children. Furthermore, the impact of gentrification in TH was explicitly mentioned by a participant as resulting in neglect of less wealthy areas:

*‘I would blatantly say the Bartlett area and the development, there has been amazing but most of those people have good money, so their access to provisions is fantastic. But* [the social landlord] *is neglecting a community like ours which is middle income or maybe even lower end I’d say, we’re suffering because they are completely neglecting these areas.’*

(TH11)

#### 3.2.5. Theme Five: Hyperlocal Safe Green Play Spaces

Families referenced activities that could be undertaken at home (e.g., toys, books, puzzles and online activities) and those that are more suited to bigger spaces outside the home, which involved physical activity. One participant explained how access to outdoor play/green area is especially valued when indoor space is limited:

*‘Outside our building we’ve got little small playgrounds. […] That’s really beneficial for all of us, especially for my kids because we don’t have a balcony and we have got no outside space, so we make use of that on a regular basis.’*

(TH13)

Participants mentioned that play and activity spaces outside the home need to be green, safe and hyperlocal (visible from the home, across or around the corner) to provide easy access and complement space available inside, together supporting children’s mental and physical wellbeing. The design of local play areas and access/views from balconies was valued:

*‘The area itself and the way it’s built with the park area and everything, it’s very nice. It’s very nice when you’ve got a good view when you’re out on the balcony and the kids are playing, the surrounding is nice.’*

(TH2)

It was also important that the various types of spaces should cater to children of different ages, and to all weather conditions. One participant explained how some of these aspects determined the family’s favourite park:

[they prefer a particular local park because it has…] *‘More green space and more peaceful and it has a playground attached to it but it also has the fence to separate it. And also I think one side is for smaller kids, the other side is for bigger kids.’*

(TH1)

In Bradford, as participants lived in terraced/townhouse and detached houses, some participants without a garden regarded the latter as an important safe space for children to play and to breathe fresh air, but those that had a garden did not always have the opportunity to use it in that way, because of various issues such as foul odours from waste in the street, as per theme 2 and 4.

#### 3.2.6. Theme Six: Unsafe Outside Local Spaces 

The impacts of anti-social behaviour and crime has been mentioned several times. Participants in TH felt a real threat towards themselves and their children from others outside their home—from drug dealing, confrontations (related to noise or misbehaviour in communal garden), and traffic speeding to knife crime and theft. In Bradford, drug dealing (especially in deserted and neglected areas such as ‘little corners’), dangerous driving, no compliance with speed limits in residential areas and proximity to busy roads, drinking next to the park, vandalism (e.g., people starting fires or breaking into the school), fights between groups of teenagers, issues with noise, racism, and bullying were mentioned as threats to children’s safety. In both areas the fear of going outside was worst at night:

*‘There’s just always drug dealing, people drinking, smoking right next to the park. Because there’s so much going on, it’s not an environment you want … you’ve constantly got to keep your hand on your child’s hand.’*

(BD9)

*‘I would say kids are bullying more and that is the reason why my kids staying most of the time in a house… would say there is nothing else what is any good for me and my kids, because we live in scary area and we are scared all the time, you never know who can bully you.’*

(BD4)

*‘We need more security basically. That’s the worst thing I would say, the safety…. When it goes dark I’m a bit scared coming home that thinking there might be someone watching…* [The police] *have* [been] *patrolling but it’s in the daytime.’*

(TH4)

Participants in TH mentioned they found protection and security inside their home whilst recognising that not everyone had that. Some said that they encouraged their children to stay at home to avoid being either a victim of crime or joining criminal or anti-social activities. In Bradford, exposure to drug dealing was a concern for parents, especially when thinking about their older kids. However, staying at home was not necessarily considered satisfactory as the often cramped homes with insufficient space could not provide all that the children need. 

Participants in both sites thought that additional security was needed, both technical (e.g., CCTV) and human including an increased police presence or patrolling from community officers. In TH some features were described as safe, these included residents-only gated communal gardens, having a balcony overlooking the communal garden, fences separating play spaces for younger and older children and having neighbours looking out for children in the park.

#### 3.2.7. Theme Seven: A Trusted Community 

For the TH study participants, the wider world was seen as untrustworthy and a risk to children’s wellbeing, as set out in the previous themes. This was further exacerbated by the anonymity of perpetrators of antisocial behaviour. When relationships worked, such as knowing people on the block and building networks of friends, people felt safe and secure and could access support. For the most part, relationships with neighbours and others in the community were talked about positively, such as the network of parents, which look after each other and their children, inside and outside the home, e.g., in playgrounds:

*‘The neighbours would check in with me and the kids.’*

(TH8)

Parents particularly valued the relationships which their children established and, as mentioned earlier, these form an important part of the decision-making around staying or leaving the area to secure a larger home:

*‘If I lose it what do the kids have? I mean we could have a nice house, they could have their individual bedrooms but for their mental health, is that really good? As opposed to going out and meeting their friends.’*

(TH9)

However, the parents needed support to come together to strengthen the network:

*‘We have great neighbours but what lacks is bringing those neighbours together, ways in which we can get people connecting more, that’s lacking in this area.’*

(TH11)

At the same time, in TH one resident suggested that more community events could be facilitated by the social landlord:

*‘We do need our community to have a get together, because in other areas I’ve seen, the community does parties for them in the area. I think the community our landlord […] should do more of this stuff.’*

(TH4)

In Bradford, one participant mentioned that living in a quiet area, where everyone knows everyone, was good for the children. Another three participants mentioned that the neighbours were helpful and caring about the kids:

*‘My family is in Pakistan and I have no one here. Here I’m alone but because my neighbours are so nice and in this area I have lots of friends. So they are helping me too much.’*

(BD7)

However, the experience of other participants was very different, one saying that there was nothing good in her area and another saying that because of language barriers they could not interact with neighbours and another saying she did not feel connected to the community but nonetheless felt safe. 

#### 3.2.8. Theme Eight: Safe and Green Spaces within Walking Distance 

Whilst theme 5 emphasises the importance of hyperlocal green outdoor spaces (e.g., playground visible from balcony), accessible green play spaces were also mentioned as important, with families also valuing well-resourced and more challenging/interesting green spaces.

*‘We have a little park that they can go and play with their friends, reconnect with other little ones if you like and just be with their peers instead of constantly being with the adults and that’s a breath of fresh air and not many people have that privilege.’*

(TH9)

Spaces that were more open and had better play equipment were preferred. Public spaces were considered important as parents could not afford to pay for private play areas (e.g., soft play areas). In both areas, there was also a call for new play equipment and play area surroundings, for use by all family members, of all ages:

*‘I’d change the play spaces, I think the play spaces need a complete rehaul, they need to redeveloped with the current times. I think the green spaces could be better, more friendly, more benches, more seating areas. They need to be more family orientated. I’d consider planting more trees.’*

(TH11)

In Bradford, local parks were described as nice, but participants thought that they did not account for the community size, as they could get overcrowded. 

#### 3.2.9. Theme Nine: Healthy and Supportive Amenities within Walking Distance 

The ability to have services and amenities for daily living within walking distance from the home was important for managing a busy family routine. Important amenities and services mentioned by participants included shops, supermarkets, schools, nurseries, hospitals, GPs, community centres, and mosques:

*‘…this area is better, I’m so happy. This area has everything nearby, the mosque and the school, the* [local community centre]*, the parks, everything is near’.*

(TH6)

Residents identified gaps, particularly practical services and amenities such as healthy food shops and local libraries, whilst noting that many businesses seemed to have closed, making the area look run down. Spaces that provided indoor activities at different times of the day and for various weathers were mentioned as important, with the cost of the activity a consideration for some parents:

*‘I don’t really feel that there is a lot for children in the area, you know, like having indoor centres, because you can’t always go to the park, it all depends on the weather…, there’s not a lot of indoor activities that are free, everything that is offered is usually it costs, so that sometimes you know, you can afford to take children to like a play centre, and other times you can’t.’*

(BD1)

Families needed indoor day spaces for play and homework, during wet and dry weather:

*‘If you actually have a real family playroom rather than just on Facebook, you have a real location. And you don’t even have to bring your stuff, that’s my space, if I’m going to do some activities just come here, if I don’t want any activities I can still come here to use it in my free time.’*

(TH1)

More after-school provisions and sports facilities were needed, including opportunities for children to build skills over time and transition between different development stages. Opportunities to encourage children of different ages to engage, and interfaith collaboration to provide activities for children, were all mentioned as needed. Finally, in Bradford, some participants identified a gap in services for children younger than 3 years old. 

Residents in both sites emphasised a need for better methods of communication to let people know about available community assets for children, or about what was going on rather than ‘last minute information’ about what was on offer and also connecting people to what is happening further afield. 

#### 3.2.10. Theme Ten: Further Away Spaces, Accessible via Transport, for Physical, Cultural and Social Development

Whilst the focus of the discussion was predominantly on the local area, some study participants cited the benefits of accessing places further away. This included visiting family and new places to encourage physical, cultural and social development. In TH, one family talked about targeting particular places with opportunities they would like for their children, such as Kew Gardens or prestigious universities.

In Bradford, participants talked about bigger parks which are not local (that they reach by car) as places they liked because they offer lots of activities and playing equipment for children. However, participants mentioned that they did not visit them very often as the distance meant that visits to such parks needed more planning (going with other friends and sorting out transport, which can be expensive):

*‘There are so many amazing things happening outside this area so if we could provide more information and support on this, that would be really good.’*

(TH1)

Some participants felt that accessing places further away was difficult due to a lack of resources and good public transport, with group discounts and direct bus routes needed.

### 3.3. Thematic Areas and Children’s Health and Wellbeing: Summary

**Figure 3:**
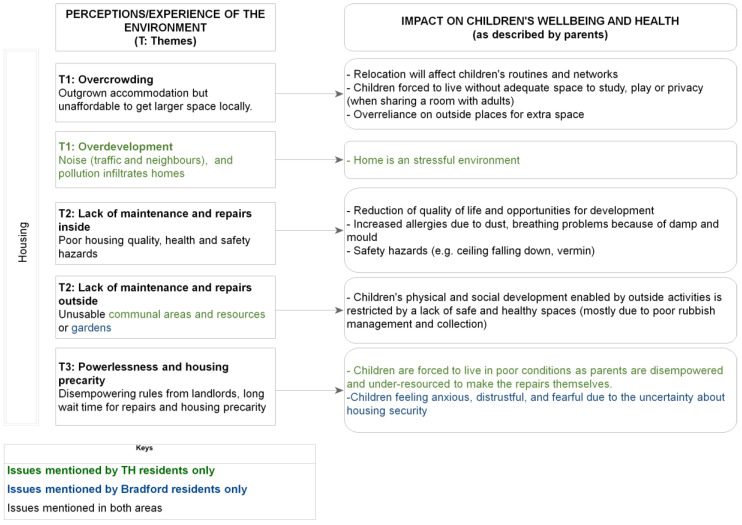
Housing themes and effect on wellbeing.

**Figure 4:**
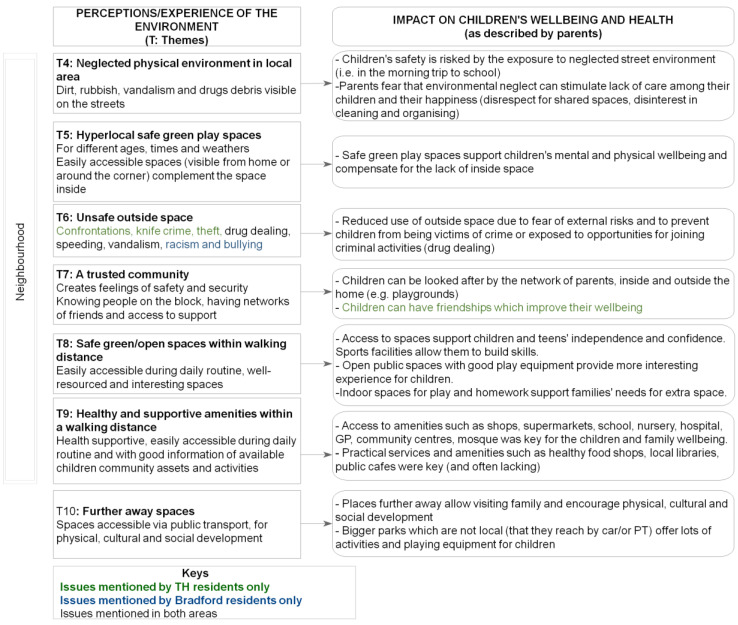
Neighbourhood themes and wellbeing.

Figure 3 and Figure 4 summarise the effects of housing and neighbourhood, respectively, on children’s wellbeing, as described by the parents during the interviews. The diagrams illustrate, from a parental lived experience perspective, how the home and the local environments can have a variety of impacts on children’s health and wellbeing. These range from aspects pertaining to physical and developmental health (e.g., respiratory problems) to health behaviours (e.g., physical activity). It should be mentioned that during the interviews, parents also frequently reported aspects that impacted their own personal wellbeing or family daily activities, as opposed to focusing exclusively on the children’s wellbeing. This could be considered as an indicator of the complex pathways through which the housing environment affects, both at an individual and household level, a family whose circumstances and wellbeing in turn deeply affect the child, as discussed in published literature [4].

**Figure 5:**
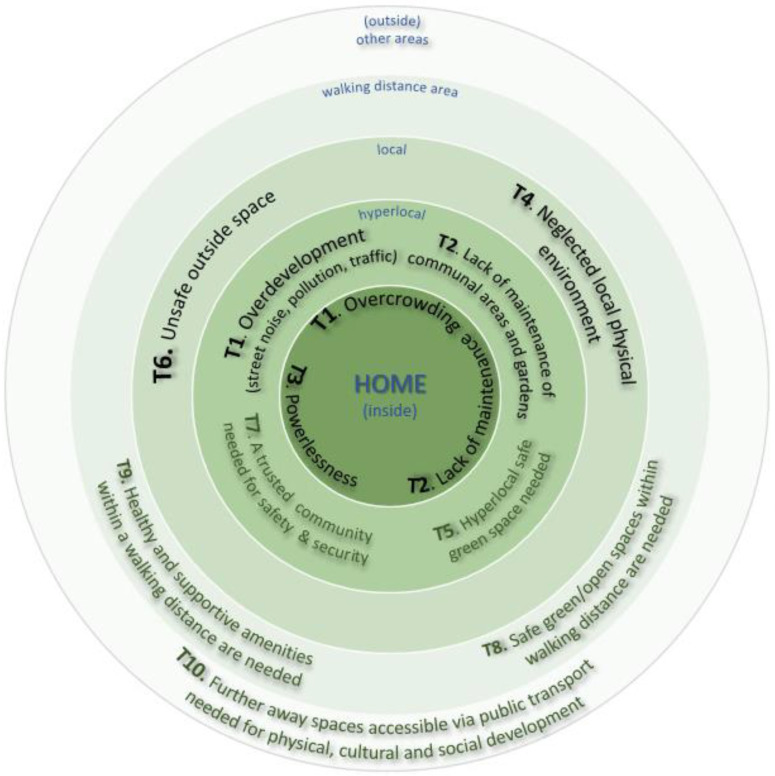
Spatial dimension of emerging themes.

We also identified a spatial dimension of the themes showing that participants described their experience of the environment at different scales going from the inside (the house) to the outside (from the hyperlocal to the parks in areas accessible via public transport). The diagram in Figure 5 illustrates this spatial dimension and also highlights how some of the themes are associated, for example the need of green and open space exists at the hyperlocal level (theme 5) and but also at the wider local-area level (theme 8).

## 4. Discussion

### 4.1. Thematic Clusters and Cross-Cutting Issues

**Figure 6:**
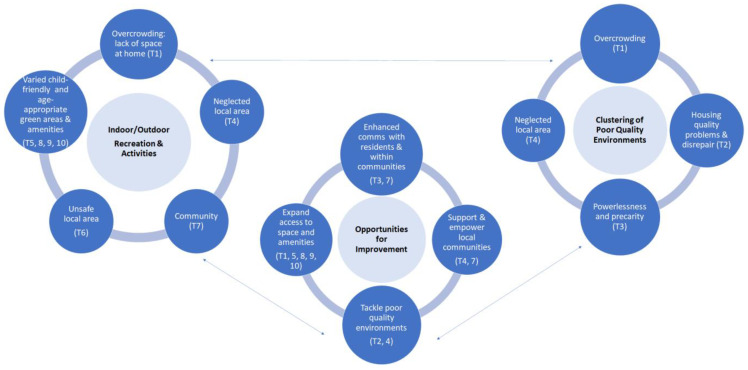
Thematic map illustrating the relationships across the ten themes via three main thematic clusters.

This study has identified ten main themes which capture the parental lived experience of children’s wellbeing as affected by the home and local environments in deprived areas in two case study settings in England. However, it is important to emphasise that these themes are interconnected and rarely act in isolation. Figure 6 illustrates the relationships across themes by mapping them within three main thematic clusters: (1) the role that indoor and outdoor places and spaces have in enabling children’s recreation and physical activity; (2) the effects of multiple quality housing/environmental issues; (3) opportunities for intervention/improvement. 

For example, overcrowding in the home environment (Theme 1)—here intended in the broad sense of space/layout not meeting the child/family’s needs—can be further exacerbated by the lack of good quality, clean and safe outdoor space for play and recreation (Themes 4 and 6). On the other hand, as other research has emphasised, feeling connected with the local community (Theme 7) can improve overall feelings of safety and belonging, thus increasing the willingness to use outdoor areas for physical activity and recreation [38,39]. However, a variety of child-friendly and age-appropriate green areas and amenities is needed to support all families, especially those with children of different age groups (Themes 5, 8, 9, 10). 

Another important aspect which emerged from the lived experience interviews was the impact of concurrent environmental problems which often act synergistically to negatively affect child and family wellbeing. For example, some participants reported experiencing both a range of overcrowding (Theme 1) and housing quality problems/disrepairs (Theme 2). The latter at times also exacerbate overcrowding issues, for instance when dampness and mould prevented a family from fully utilising an area within their home. Some families reported experiencing disrepair and neglect both within their home and in their local surroundings (Theme 4), which not only affected aspects of physical health or health behaviours, but potentially also mental health via feelings of neglect (e.g., local authorities favouring more affluent communities and more lucrative local developments, Theme 4) and powerlessness (Theme 3). These aspects were also especially apparent in various examples of unresponsiveness, delays and general dissatisfaction with the landlord’s and/or the local authority’s approach to reports of disrepair/problems in the home, or antisocial behaviours and environmental issues locally (e.g., collection of waste). Previous research has indicated that psychosocial factors such as satisfaction with the landlord can affect the mental wellbeing of adults living in deprived areas [13]. For those participants experiencing a multitude of significant problems within their dwellings, the need to address them was the priority, over and above access to good quality outdoor space. 

On the other hand, various opportunities for change were identified during the interviews. These included:

1. The need to expand access to space and amenities, for example, by providing greater affordable access to storage space (thus helping with overcrowding problems, Theme 1) and increasing the variety of play/green/recreation areas (Themes 5, 8, 9, 10).
2. Tackling poor-quality environments and/or disrepair inside and outside the home (Theme 2 and 4).
3. Better communication with residents and within the local community. For example, some participants mentioned the need to better communicate child-friendly initiatives run by local organisations or communities (Theme 7). Furthermore, the lack of communication with and involvement of residents in activities related to the prioritisation and fixing of disrepair or hazards inside and outside the home further compounded the direct effect of such problems, by disempowering and belittling residents (Theme 3).
4. Supporting and empowering local communities to ‘take ownership’ of local places, spaces and initiatives. Some residents emphasised the value of the local communities in creating places and activities which support various aspects of children’s wellbeing (Theme 7) and also in increasing feelings of social cohesion and safety in response to anti-social behaviours (Theme 6).

Overall, the ten themes identified in this study, and the relationships presented in Figure 6, demonstrate the complex web of interactions through which the housing and the neighbourhood physical and social environments impact the wellbeing of children and their families. The exponential, rather than additive, nature of these exposures have been emphasised by Swope and Hernandez [17], whose conceptual model of housing as a determinant of health equity includes factors extensively discussed in this study (housing and context). Whilst this study did not explicitly seek to draw out aspects related to the other two pillars of Swope and Hernandez’s model (i.e., affordability and residential stability), it is important to acknowledge their critical role and indeed their relationships to the themes discussed in this study. For example, some participants reported that affordability was often related to overcrowding (i.e., cannot afford larger home) and/or to the importance of social ties (i.e., thus preventing families to move in a more affordable area). In one case in Bradford, the reporting of a housing quality problem had resulted in the resident being evicted by the landlord, who in turn had decided it was more cost-effective to sell their property than repair it for rental purposes. In TH, a resident reported that since they were classified as ‘homeless’, they were given little choice in their housing allocation and thus ended up being placed in a property which was too expensive for them. 

### 4.2. Strengths and Limitations

This exploratory study addresses a relatively under-researched field, i.e., understanding how children’s health in deprived areas may be affected by the complex systems of interactions between the home and local environments. Whilst previous research has identified various risk factors for child health and inequalities related to the home and/or neighbourhood environments, this study adds to the body of evidence by examining these factors and their interrelationships through the lived experience lens, which is an important dimension for the identification of suitable interventions and for helping make the case for change [17]. Whilst the evidence presented in this study could have practical implications for urban design, further consideration will be needed to establish how our findings relate to relevant urban planning principles and evidence, such as those originally set out by Jane Jacobs [40] and further more recent discourse [41,42]. A further strength of this study was its ability to involve seldom-heard communities, which was possible via partnering with community-embedded organisations whereby pre-established trust enabled reflective, nuanced and open conversations with participants. In Bradford, there was also an opportunity to conduct interviews in participants’ first language (Urdu, Punjabi and Roma (Slovakian)), which further widened opportunities for inclusion. On the other hand, however, the completion of the online questionnaire proved hard for some participants, especially those with limited digital means and/or relatively poor knowledge of the English language, although an offer to assistance with the online survey was made, with some participants opting for an interviewer-administered completion via phone instead. Future studies should thus consider how to further widen participation, especially for the questionnaire component. It should be especially emphasised that participants within the targeted communities had limited time availability, whereby some who had initially expressed an interest found it difficult to allocate time to the interview. Future studies could consider ways to free up participants’ time (e.g., offering childcare/activities as options during the interview). Whilst this study’s primary goals did not include the systematic identification of differences between the two settings (i.e., TH and Bradford), it should be acknowledged that such differences (e.g., in Figure 2, Figure 3 and Figure 4) could be related to a combination of factors, including variation in housing types and/or built density between the two areas, as well as a greater diversity in the Bradford sample in terms of ethnicity and housing tenure. 

The interviews were conducted during the COVID-19 pandemic and thus some aspects reported by participants may have been exacerbated by successive lockdowns and/or a reduction in services offered by landlords or local authorities. However, participants were asked if matters they had discussed during the interviews had changed during the pandemic. By and large, most issues had not dramatically changed in nature, although some had changed in severity or frequency. Indeed, this emphasises how, as a result of the pandemic, many issues experienced by residents in deprived communities have been aggravated, therefore requiring urgent coordinated action to address them. 

## 5. Conclusions 

This study provides new insights into how interactions between housing and the local environment impact children’s health and wellbeing, through the lens of the lived experience of parents in areas with high levels of child poverty in England. Findings emphasise how the wellbeing of individual children, their parents and the whole family can be deeply affected by these relationships, which often occur as a spatially patterned ‘cluster’ of problems, some of which act through feedback loops to reinforce their impacts on various dimensions of physical, mental, developmental and social health. 

The evidence presented in this research emphasises how environmental quality issues within and outside the home, compounded further by delays in repairs and reduction in service standards (partly due to the COVID-19 pandemic), as well as issues of affordability (which can only become worse in light of the cost of living crisis), are likely to deeply affect an entire generation of disadvantaged children whose parents feel disempowered, neglected and often isolated when trying to tackle various dimensions of inequalities. The importance of a variety of child-friendly, safe and age-appropriate green and play areas/activities within and outside the home was a theme which complemented quite consistently the need for decent homes, free from hazards and with enough space for daily living, including home learning, play and socialisation. Interventions which can improve access to space and services, as well as the quality of housing within the context of the local environments, are therefore urgently needed. 

The research also emphasised the role of the social environment as a support system for children and their families, which enables overcoming some of the problems within the local or home environments, or conversely as a deterrent, which prevents families from utilising some areas deemed as unsafe or neglected. Some participants also emphasised the importance of communication channels related to local activities/services and of feeling valued as well as empowered to contribute to decisions pertaining to repairs or upkeep within and outside their homes. Therefore, initiatives which can support and empower individuals and local communities are essential, especially those which can help understand local priorities and opportunities for action, since overall resources available to individuals and organisations are limited in the light of the growing cost of living crisis caused by factors such as the COVID-19 pandemic, rising fuel costs and worldwide unrest. 

Many of the aspects highlighted in this study, including the opportunities for improvement, resonate with various activities which the UK Government has initiated to reform social housing, including the Social Housing White Paper (17 November 2020), and the Levelling Up White Paper (2 February 2022) which in turn had a commitment to bring forward a Social Housing Regulation Bill. These various initiatives aim to address aspects such as: ‘*empower residents to support them in engaging with and holding their landlords to account*’ and ‘*ensure good quality, decent homes and neighbourhoods*” but also “*Strengthen the consumer standards social landlords must meet and create a strong, proactive regime to enforce them*’ [43]. This study emphasises the importance of all these aspects for children’s wellbeing and also highlights the importance of working in partnership with local communities—particularly when identifying priorities and criteria for improvements. Furthermore, the study also highlights how more responsive and proactive systems are needed to support tenants in both the social and the private sector experiencing multiple levels of disrepair, environmental hazards and anti-social behaviour. 

Although our research aimed to focus on children’s health and wellbeing, the findings also highlight that an individual child’s health/wellbeing is inextricably connected to the needs and routines of other family members and that children’s needs related to the home and neighbourhood environments vary quite considerably as they grow. In this sense, the design and retrofit of homes and local neighbourhoods should consider more systematically how to embed variety and flexibility to better cater for the changing needs of individual children as they grow, as well as of families with children of different ages. 

Whilst this is an exploratory study based on a relatively small sample, it included parents living in two local authorities in England with high levels of child poverty but differing in aspects such as population density and housing costs, whereby the themes identified in this research were similar across the two areas. These emphasised the complex system of physical and psychosocial interactions through which housing, and neighbourhoods, impact children’s health, highlighting the urgent need for action. Future studies should include holistic approaches to child wellbeing, including measurements of physical, mental, developmental and social health parameters in relation with housing and neighbourhood environmental conditions. Further research is needed on how communities and key stakeholders should be supported in identifying and agreeing upon priorities for interventions within the local area, which are both inclusive and target those most deeply affected, whilst leveraging the positive interactions between themes identified within this study and avoiding at the same time any unintended consequences of single-focus policies. 

## Figures and Tables

**Table 1:** Participant self-reported demographics, wellbeing score (adults), accommodation details and coping with finances (data from fully completed questionnaires only).

Participant ID	Site	Age	Gender	Ethnicity	WEMWBS Cat *	Property Type	Landlord	N Adults	N Children	Enough Living Space? ^3^	People per Bedroom	Outdoor Space? ^4^	Coping with Finances
TH1	TH	35 to 44	Female	Prefer not to say	High	High-rise Flat ^1^	NA (owned property)	2	1	No	1.5	No	Prefer not to answer
TH2	TH	35 to 44	Female	Asian or Asian British	Low	Low rise purpose-built flat ^2^	Local authority	2	2	No	2	Yes	Finding it very difficult
TH4	TH	25 to 34	Female	Asian or Asian British	Low	High-rise Flat ^1^	Housing association	2	4	No	3	Yes	Doing all right
TH5	TH	35 to 44	Female	Asian or Asian British	High	High-rise Flat ^1^	Local authority	3	1	No	2	No	Prefer not to answer
TH6	TH	Prefer not to say	Female	Asian or Asian British	High	Low rise purpose-built flat ^2^	Housing association	2	4	No	3	Yes	Prefer not to answer
TH7	TH	25 to 34	Female	Asian or Asian British	Low	0	Private landlord	2	3	No	2.5	No	Don’t know
TH8	TH	25 to 34	Female	Asian or Asian British	Low	Low rise purpose-built flat ^2^	Local authority	1	2	No	3	No	Doing all right
TH9	TH	35 to 44	Female	Asian or Asian British	High	Low rise purpose-built flat ^2^	Housing association	2	3	No	2.5	Yes	Just about getting by
TH10	TH	35 to 44	Female	Asian or Asian British	Low	Terraced/Townhouse	Housing association	3	1	Yes	1.3	Yes	Doing all right
TH11	TH	35 to 44	Female	Asian or Asian British	Low	High-rise Flat ^1^	Housing association	2	2	No	2	No	Finding it quite difficult
TH12	TH	35 to 44	Female	Asian or Asian British	Low	Low rise purpose-built flat ^2^	Local authority	3	3	No	2	Yes	Finding it quite difficult
TH13	TH	35 to 44	Female	Asian or Asian British	High	Low rise purpose-built flat ^2^	Housing association	2	2	Yes	2	No	Doing all right
BD1	BD	25 to 34	Female	Asian or Asian British	Low	Terraced/Townhouse	NA (owned)	1	1	Yes	0.5	Yes	Finding it very difficult
BD2	BD	25 to 34	Male	White	High	Detached	Private landlord	2	1	No	1	Yes	Just about getting by
BD3	BD	35 to 44	Male	Other (please specify)	Low	Terraced/Townhouse	Private landlord	2	2	No	2	No	Just about getting by
BD4	BD	25 to 34	Female	Other (please specify)	Low	Terraced/Townhouse	Private landlord	1	2	No	1.5	No	Finding it quite difficult
BD5	BD	18 to 24	Female	White	High	Other	Housing association	2	2	Yes	2	Yes	Living comfortably
BD6	BD	25 to 34	Female	Asian or Asian British	Low	Detached	Private landlord	1	1	No	2	No	Finding it very difficult
BD7	BD	25 to 34	Female	Asian or Asian British	High	Other	Private landlord	1	2	Yes	1	Yes	Don’t know

TH = Tower Hamlets; BD = Bradford; ^1^ Building over 6 storeys; ^2^ Building with 6 storeys or less; * Based on the Warwick-Edinburgh Mental Wellbeing Scale (WEMWBS) where high approximate higher than average wellbeing and low being lower than average wellbeing; ^3^ ‘Do you think there is enough living space for you and your family?’; ^4^ ‘Does your home have an outdoor space which you and your family can use?’.
